# How do MNEs shape their international strategic alliances to facilitate higher alliance performance? Absorptive capacity as an antecedent

**DOI:** 10.3389/fpsyg.2022.955117

**Published:** 2022-10-11

**Authors:** Zhong Chen, Langping Zheng, Michael Yao-Ping Peng, Lijin Shao

**Affiliations:** ^1^Research Center of Open Economics and Trade, Fuzhou University of International Studies and Trade, Fuzhou, China; ^2^School of Business, Xiamen Institute of Technology, Xiamen, China; ^3^Business School, Foshan University, Foshan, China; ^4^School of Economics and Management, Fujian College of Water Conservancy and Electric Power, Yongan, China

**Keywords:** absorptive capacity, international strategic alliance, international performance, multinational enterprises, structural equation modeling

## Abstract

The international strategic alliance in international marketing is international social network to realize superior performance. This requires firms to increasingly consolidate international relationship with foreign partners. Knowledge acquisition and integration by absorptive capacity (AC) as an antecedent for international strategic alliance is understudied. This study aims to explore the relationship between AC and international strategic alliance, and their impact on the international performance of multinational enterprises (MNEs). This study empirically verifies the research framework from 223 Taiwanese MNEs. In terms of structural model, researchers conduct PLS-SEM to verify the hypotheses in this study. The results confirm previous studies that indicate a positive relationship among AC, international strategic alliance, and international performance. The results also indicate that international explorative and exploitative alliance fits mediate the relationship between AC and international performance.

## Introduction

Following the tendency of globalization, to pursue large economic scope, enterprises tended to expand their markets or transfer their factories to low labor-cost countries (Sethi and Guisinger, 2002; Knight and Liesch, 2016; Spyropoulou et al., 2018). With regard to the nature of the foreign market, multinational enterprises (MNEs) are confronted with more challenges from the international situation than the local situation (Sethi and Guisinger, 2002; Cavusgil and Knight, 2015; Gerschewski et al., 2018; Sadeghi et al., 2018), especially in service and/or information technology industries. MNEs are based on the fundamental assumption that they may increasingly adjust operation conditions and resource distributions during the internationalization (Cavusgil and Knight, 2015; Spyropoulou et al., 2018) and lack sufficient ability and knowledge to deal with challenges in international competitions (Knight and Liesch, 2016; Johanson and Vahlne, 2017; Khan and Lew, 2018). Through the acquirement of international knowledge, MNEs can better leverage learning and innovation capability and thus improve international performance.

Recently, many scholars have been paid attention to strategic alliance, especially in strategic and network management (Rui et al., 2016; Delery and Roumpi, 2017; Harrigan and Diguardo, 2017). In domestic industrial environment, firms acquire valuable knowledge and intelligence through various strategic alliances to improve performance, competitive advantage, innovation, new product development, etc. However, the above-mentioned is the domestic perspective of strategic alliance, but it is a lack of understanding from the internationalization perspective (Li and Zhou, 2010). MNEs not merely need to face the competition from the domestic industry, but also deal with the challenges brought from the foreign competitors' behaviors and the heterogeneity of foreign markets, which emphasizes on the significant role of international strategic alliance (Kwak et al., 2018). The international strategic alliances can be regarded as a cooperation mode between MNEs under international mobility and connection, linking goals and missions of MNEs to enhance the resource usability and the autonomy management of organizations (Nielsen and Gudergan, 2012; Li et al., 2013; Ho and Wang, 2015). Most studies of international strategic alliances focus on understanding how MNEs improve performance, but few studies further discuss whether MNEs can generate activities about knowledge complementarity and knowledge spillover to enhance their performance, together with members in alliances after entering a specific alliance. According to the relationship marketing theory, the configuration of international strategic alliance portfolios has a vague relationship with performance, which is a theoretical gap. MNEs' management of each alliance and alliance portfolios will indirectly affect the development of its internal organizational capabilities (Rezende da Costa et al., 2018; Pereira et al., 2021). However, this was seldomly mentioned in resource-based views (Li et al., 2013), dynamic capability views (Abu-Rumman et al., 2021), knowledge-based views, and organizational learning views. Thus, the performance of international strategic alliance can be regarded as a measure of the actual outcomes of knowledge spillover, sharing, and integration among alliance members. The high alliance performance signifies a high degree of knowledge integration among alliance members, which is conducive to MNEs providing products and services that can meet the needs of foreign customers in the international market and product development process.

According to organizational learning theory, in order to overcome the boundaries among organizations, firms mainly access or learn new knowledge from business network and then integrate the new knowledge with the existing knowledge base in order to develop specific-based knowledge that belongs exclusively to the organization (Liao et al., 2016). From this perspective, it implies that MNEs must be equipped with specific and enough knowledge base and processing capability to intensify the linkage with international strategic alliance. These processes are referred to as absorptive capacity (AC) (Sun and Anderson, 2012; Larrañeta B. et al., 2016). The development of AC reinforces, complements, and focuses on the knowledge base possessed by the MNEs (Lane et al., 2006), facilitating the fit with international strategic alliance, and thus promoting the development of the MNEs' international performance. In international strategic alliance, by virtue of the cooperation with other partners, MNEs are able to strengthen their acquisition and absorption of knowledge, improve their innovation in products, and solidify the knowledge base by their enhanced absorptive ability (Rezende da Costa et al., 2018; Li and Reuer, 2022). Thus, the promotion of AC enables MNEs to leverage advantage of obtaining, assimilating, and applying the knowledge to strength fit of international strategic alliance, and then to improve MNEs' innovativeness and proactiveness (Atuahene-Gima and Murray, 2007). Based on the above arguments, this study aims to explore the relationship among AC, international strategic alliance, and alliance performance.

According to discussions on AC, scholars have proposed multiple perspectives of operationalization. Specifically, several scholars claim that AC is a series of knowledge processing activities, including knowledge acquisition, assimilation, transformation, and exploitation (García-Villaverde et al., 2018). AC is classified into potential AC (PAC) and realized AC (RAC) by other scholars (Kang and Lee, 2017; Flor et al., 2018; Limaj and Bernroider, 2019). But these views have something similar. And AC is considered as a high-order dimension, which consists of various measuring variables (Kang and Lee, 2017; Flor et al., 2018; García-Villaverde et al., 2018; Limaj and Bernroider, 2019). This study adopts reconceptualization of AC proposed by Kang and Lee (2017) and Flor et al. (2018), which distinguishes between PAC and RAC (Peng, 2022). Based on prior arguments, there are three reasons to discuss why the AC plays a key role in our research framework. Firstly, although most of previous literatures contributed to provide more insights on our understanding of AC concept, there is still a lack of empirical studies exploring formation and development of AC specifically (Kang and Lee, 2017; Limaj and Bernroider, 2019). Additionally, there are few researches to explore the role of AC in the context of MNEs, which is critical in discussing how MNEs foster superior AC to adapt to rapidly dynamic market environment with scarce resources. Thirdly, there is a lack of empirical research to verify multiple relations of PAC and RAC, which is particularly important in the context of MNEs (Heavey et al., 2015; Broersma et al., 2016; Rafailidis et al., 2017). However, when MNEs distinguish external knowledge resources, the development of PAC and RAC is conducive to obtaining knowledge and resources that are more beneficial for the enhancement of core competitiveness, and approaching alliances that are equipped with homogeneous knowledge so as to improve marketing performance. Thus, this study aims to explore the impact of PAC and RAC on international strategic alliance fit in the context of MNEs.

This study aims to explore the development of AC of MNEs based on the organizational learning view in the international context, which provides several contributions. First of all, in terms of theoretical contribution, this study proposes a complete research framework combined with the relationship marketing theory and organizational learning view in order to verify the importance of the configuration of international strategic alliance and enrich the connotation and significance of the international marketing perspective in the context of MNEs. Second, this study discusses the different types of international strategic alliances and explores MNEs' selection of and entry into appropriate alliances in international markets from the perspective of capability development in order to enhance the performance and competitiveness of MNEs in the international environment. Third, compared to previous studies that have discussed the impact of the selection of strategic alliance on capabilities and performance, this study puts emphasis on the relevance between MNEs' capability development and different types of alliance fit, and how to obtain more fit alliance in order to reduce the risks brought by unfitness.

## Theoretical background and hypotheses

### Organizational learning and alliance performance

Organizational learning theory has appealed to most scholars involved in management theory over the past two decades, and the theory has been abundant in knowledge domain in different areas (Liao et al., 2016; Peng, 2022). According to scholars, knowledge plays a significant role in firm capacity and boundary. For the sake of limitation and replication prevention of competitors, most firms are engaged in externally new knowledge acquisition, or new knowledge learning from partners, as well as integration of novel and existing knowledge, resulting in efficiency improvement of internal process or development acceleration of new products (Liao et al., 2016). Based on organizational learning, new knowledge acquisition becomes more and more, and existing knowledge gets more frequently updated, enabling MNEs optimize their learning and apply their knowledge base to strategic alliances (Liao and Hu, 2007; Huang and Yu, 2011; Larraneta E. et al., 2016).

The literature on the performance of strategic alliance from the organizational learning perspective has been growing. Most of them have been empirically supported, no matter from MNEs context, the influence of type of international strategic alliance on alliance performance (Li and Zhou, 2010; Rui et al., 2016; Pereira et al., 2021; Li and Reuer, 2022). However, the persistence of alliance performance (Harrigan and Diguardo, 2017), and how MNEs in various international strategic alliance achieve higher alliance performance (Kwak et al., 2018) were rarely discussed. When the alliance performance is improved, the alliance can extend more knowledge overflow effects and motivate MNEs in it to pay more attention to the international cooperation with each other and to create an environment for mutual learning and knowledge sharing, so that they can enhance their performance in the alliance during the process of knowledge derivation, combination, expansion, and creation. Thus, the alliance performance under international context also needs to be incorporated with organizational learning to further understand how the development of MNEs' AC indirectly influences alliance performance (Li and Zhou, 2010; Peng, 2022).

### International strategic alliance

Strategic alliance refers to a cooperation agreement reached between firms in the industry in order to achieve a certain common goal. Members of the alliance will follow a common norm and reach consensus to some extents (Robson and Katsikeas, 2005; Nielsen and Nielsen, 2009). More specifically, strategic alliances are referred as interfirm cooperative agreements in which firms are joint to develop long-term product cycle and establish market position with their partners (Bakermans-Kranenburg et al., 2005). Previous studies on strategic alliances have filled many research gaps from the perspective of organizational learning and knowledge transfer, including: (1) to manage knowledge in strategic alliances, (2) to transfer knowledge across partners, (3) to acquire knowledge from the parents by the joint venture itself, and (4) to collaborate knowledge per se develops over time and impacts collaborative outcomes (Nielsen and Nielsen, 2009; Ho and Wang, 2015; Pereira et al., 2021; Zhou et al., 2022). It can be found from these gaps that knowledge learning and transfer within the alliance has a strong strategic significance for the competitiveness of firms (Nielsen and Nielsen, 2009).

However, an increasing number of studies have extended the concept of local strategic alliance to the international strategic alliance, in which theories such as resource-based views, dynamic capabilities, organizational learning, and corporate internationalization are combined, and an interdisciplinary research field is formed. Over the past two decades, scholars have summarized three research trends, including theoretical vs. empirical, outcome vs. process, and technology-based vs. marketing-based. In the context of technological innovation and globalization, international strategic alliances focus more on the stability, growth, and long-term development of international business organizations than local strategic alliances (Bakermans-Kranenburg et al., 2005; Robson and Katsikeas, 2005; Peng and Shao, 2021). The international strategic alliances can be regarded as a cooperation mode between firms under international mobility and connection, linking goals and missions of firms to enhance the resource usability and the autonomy management of organizations (Nielsen and Gudergan, 2012; Li et al., 2013; Ho and Wang, 2015; Li and Reuer, 2022). Robson and Katsikeas (2005) also pointed out that international strategic alliances are foreign investment activities that involve a continuous process of business activity, and formative issues of intra-partner characteristics and inter-partner fit are emphasized (Pereira et al., 2021). Therefore, the types of alliance will also be different due to different partner characteristics and fit modes. Nielsen and Gudergan (2012) divided ISA into international exploration alliances and international exploitation alliances by referring to Rothaermel's (2001) and Lavie and Rosenkopf's (2006) perspective. They defined international exploration alliances as primarily engaged in upstream activities of value chain to shape innovative concept and create new knowledge jointly; international exploitation alliances instead focus on downstream activities of value chain to provide required product and service for international customers, such as marketing and distribution. The classification of such two kinds of alliances is like the classification of DICs by ambidexterity and does not consider the demands of different alliance characteristics on knowledge attributes, strategic behaviors, and innovation (Peng and Shao, 2021). Thus, international companies will join one of the ISAs based on different organizational structures, capabilities, strategies, processes, cultures, and purposes. International exploitation alliances provide internationalized firms with more exploitation knowledge that increases the productivity and efficiency of capital and assets through standardization, systematic cost reductions, and improvements to existing technologies, skills, and capabilities (Nielsen and Gudergan, 2012). Similarly, international exploration alliances provide internationalized firms with more exploration knowledge that facilitates discovery of new opportunities for wealth creation and above-average returns through innovation, new capabilities, and investments in AC (Nielsen and Gudergan, 2012).

MNEs in international exploration alliances establish a mutual learning culture through the contact and interaction with firms of other countries based on a stable and strong cooperative relationship (Nielsen and Nielsen, 2009; Ho and Wang, 2015; Li and Reuer, 2022). Such informal agreements give international firms the opportunities to access the resources of knowledge innovation from the alliance network, while international firms in the alliance can use joint problem solving, joint new product development, and joint R&D investment projects (Bakermans-Kranenburg et al., 2005) to accumulate more internal innovation capabilities (Li et al., 2013). Scholars indicated that the higher efficiency of MNEs in pursuing close strategic relationships (Nielsen and Nielsen, 2009) will help them to create opportunities of collaboration with other foreign partners and gain the knowledge and information required to strengthen the value of innovation capability, thereby further enhancing the innovation and new product development (Robson and Katsikeas, 2005). Besides, MNEs will be able to understand the operating model of benchmarking enterprises and the process of knowledge processing through the strategic relationship among the members of international exploration alliances, so that the members develop the skills to detect external knowledge and further correct the internal routines, process, and production efficiency (Nielsen and Gudergan, 2012; Li et al., 2013). The process of external learning promoting internal learning is conducive to MNEs to strengthen their basic capabilities and viability, as the basis for business growth (Nielsen and Gudergan, 2012; Ho and Wang, 2015; Pereira et al., 2021). Nielsen and Nielsen (2009) pointed out that international inter-firm relationships with innovative learning can enhance the understanding of tacit knowledge of both firms, improve their inadequacies in knowledge application, strengthen the link between relationships and knowledge, and further improve the ability to apply innovation. Thus, the study makes hypotheses as follows:

*H1: International exploration alliance has a positive impact on alliance performance*.

As for the knowledge-based view, MNEs are considered as repositories of knowledge and competencies, and knowledge acquisition is significantly attached to the learning-based perspective, thus contributing to the development of firm-specific capabilities. According to both knowledge-based and organizational learning theories, it is considered that the characteristics of the knowledge may have an impact on the ease (or difficulty) of learning and knowledge transfer. Inkpen (2002) identified five antecedents of alliance learning: (1) learning partner characteristics; (2) teaching partner characteristics; (3) knowledge characteristics; (4) relationship factors; and (5) alliance form and calls for work that integrates the diverse categories and establishes some causal links across the variables (Ho and Wang, 2015). MNEs in international exploitation alliances are committed to learning and absorbing explicit knowledge that can immediately improve the internal operating processes. Compared with MNEs in international exploration alliances, they pay more attention to diversified relationship connection, such as structural hole and weak ties mentioned by scholars. In the process of knowledge transfer or knowledge learning, MNEs also learn how to create value by knowledge while accumulating knowledge assets (Nielsen and Nielsen, 2009; Nielsen and Gudergan, 2012). For one thing, MNEs obtain the needs of customers in the international market and the information of competitors from external sources of knowledge so as to provide products and services more efficiently. For another thing, MNEs absorb more diverse sources of knowledge from alliances, which help to enhance the related activities and capabilities of innovation and further improve the development of new products or service. On this basis, the hypotheses are developed as follows:

*H2: International exploitation alliance has a positive impact on alliance performance*.

### Absorptive capacity

Few studies have been conducted on subjects about the AC, such as how to acquire innovative knowledge from the external environment, how to replace firms' activities for knowledge creation, and how to enhance innovation activities and performance through combining existing knowledge base with external knowledge (Cohen and Levinthal, 1990; Wu et al., 2020; Peng, 2022; Zhou et al., 2022). Lane et al. (2006) made definition of AC, and it refers to the capacity of firms in which external knowledge can be effectively used in three consecutive processes, including: (1) to make identification and understanding of potential valuable knowledge outside the firm by means of exploratory learning; (2) to absorb new valuable knowledge by means of transformative learning; and (3) to apply the knowledge which is absorbed to new knowledge and commercial consequence creation by means of exploitative learning (Peng and Lin, 2019; Peng, 2022; Zhou et al., 2022).

According to the arguments of Escribano et al. (2009), AC relies on the current knowledge assets of firms, and the emphasis is put on the recognition, digestion, and utilization of new knowledge. Thus, the knowledge assets are mostly embedded in products, procedures, and personnel (Peng and Lin, 2019). In regard to conceptualization, most scholars illustrated that AC is a latent construct of multidimension (Zahra and George, 2002; Flattena et al., 2011; Kang and Lee, 2017; Limaj and Bernroider, 2019; Peng, 2022; Zhou et al., 2022). For the first-order level, AC contains a series of four capabilities, which includes acquisition, assimilation, transformation, and exploitation. The capabilities of acquisition and assimilation form the PAC. The capabilities of transformation and exploitation make up the RAC (Zahra and George, 2002; Limaj and Bernroider, 2019; Wu et al., 2020). Generally, PAC is a process of acquiring and digesting external knowledge, and creating new knowledge through internal process, while RAC is a process of converting internal knowledge and using it to cope with environmental changes (Zahra and George, 2002; Flatten et al., 2011; Limaj and Bernroider, 2019). Namely, PAC and RAC can be conceptualized as second-order dimensions of AC (Camisón and Forés, 2010; Peng, 2022; Zhou et al., 2022).

Previous studies used the AC lens to explain innovation, performance, or competitive advantage through various theoretical framework and perspectives, such as social capital (García-Villaverde et al., 2018), organizational culture (Limaj and Bernroider, 2019), and innovation (Kang and Lee, 2017; Flor et al., 2018). Specifically, organizational theory contributes to highlighting antecedents, reasons, and insights under which AC creates value and advantage (Zahra and George, 2002; Jansen et al., 2005; Lane et al., 2006; Limaj and Bernroider, 2019). In general, PAC is used to acquire and digest knowledge gained from outside, and then to create new knowledge through internal process. In the process of input-process-output, the quality and amount of knowledge must be established. Therefore, MNEs with well-developed PAC will be able to facilitate the connections with international exploration and exploitation alliance based on acquisition of more new, implicit, and tacit knowledge and information with low repeatability (Liao et al., 2016). When the knowledge is introduced during the internal knowledge integration and knowledge creation, MNEs will be able to cooperate with members in international exploration and exploitation alliance to improve efficiency of knowledge transferring and knowledge creation, thereby enhancing their innovation and market operation capability. Therefore, the relevant hypotheses are made as follows in this study:

*H3: Potential absorptive capacity has a positive impact on MNEs' fit with international exploration alliance*.

*H4: Potential absorptive capacity has a positive impact on MNEs' fit with international exploitation alliance*.

Nevertheless, RAC does not conform to all units of an organization that diverse knowledge is needed for new ideas and innovative products production in the construction and accumulation of RAC (Escribano et al., 2009; Broersma et al., 2016). If there is no RAC, knowledge transfer from other units is not available (Lane et al., 2006). And the internationalized process of firms is a kind of organizational learning. It is necessary to integrate knowledge and information in the international market with RAC, so as to facilitate MNEs in making a clear strategic position and confirming demands of global customers, thus making exploitation capability improvement and assessment of the international exploration and exploitation alliance (Eisenhardt and Martin, 2000; Rothaermel and Alexandre, 2009; Morgan et al., 2010). Furthermore, vicissitudes in the environment of international market are much more fierce than that in local market. Enough information and capacities are needed for MNEs in adapting to competitor and market changes. With high degree of RAC, MNEs are enabled to adjust internal knowledge in line with changes in external environment put forward specific strategic behaviors, obtaining a leading position in strategies (Liao et al., 2016). Besides, RAC is also proposed to be capable of innovative behavior promotion of MNEs and process acceleration of knowledge creation with members of exploration and exploitation alliances, thus leading competitive activities of competitors (Limaj and Bernroider, 2019). Therefore, the relevant hypotheses are made as follows in this study:

*H5: Realized absorptive capacity has a positive impact on MNEs' fit with international exploration alliance*.

*H6: Realized absorptive capacity has a positive impact on MNEs' fit with international exploitation alliance*.

PAC and RAC are critical drivers for enhancing knowledge integration and creation. PAC can increase the reliability and productivity of knowledge, whereas RAC can strengthen organizational capabilities to integrate new/external knowledge to the internal knowledge base, thereby enabling MNEs to respond to competitors' behaviors (Koryak et al., 2018). Most of previous studies have verified the impact of RAC and PAC on objective variables, such as innovation (Kang and Lee, 2017; Flor et al., 2018; Limaj and Bernroider, 2019), learning sustainability (Riikkinen et al., 2017), and new product development and performance. The effective interaction between PAC and RAC will facilitate MNEs to achieve the profitability of existing markets continually and reduce the risk of over-emphasizing PAC or RAC (Heavey et al., 2015; Broersma et al., 2016; Vahlne and Jonsson, 2017; Luca et al., 2018; Spyropoulou et al., 2018). This study further discusses the relationship of hierarchical order between the two capacities, i.e., PAC has a positive effect on RAC in a context of MNEs (Riikkinen et al., 2017). Portfolios are diversified by MNEs with abundant routine and profitable projects, as well as projects that are high-risk and ground-breaking, on the basis of various strategic objectives (Rafailidis et al., 2017), which makes MNEs available in current strengths leverage and risk embrace so as to drive future market opportunities (Spyropoulou et al., 2018). Thus, this study proposed the following hypothesis:

*H7: Potential absorptive capacity has a positive impact on Realized absorptive capacity*.

**Figure 1:**
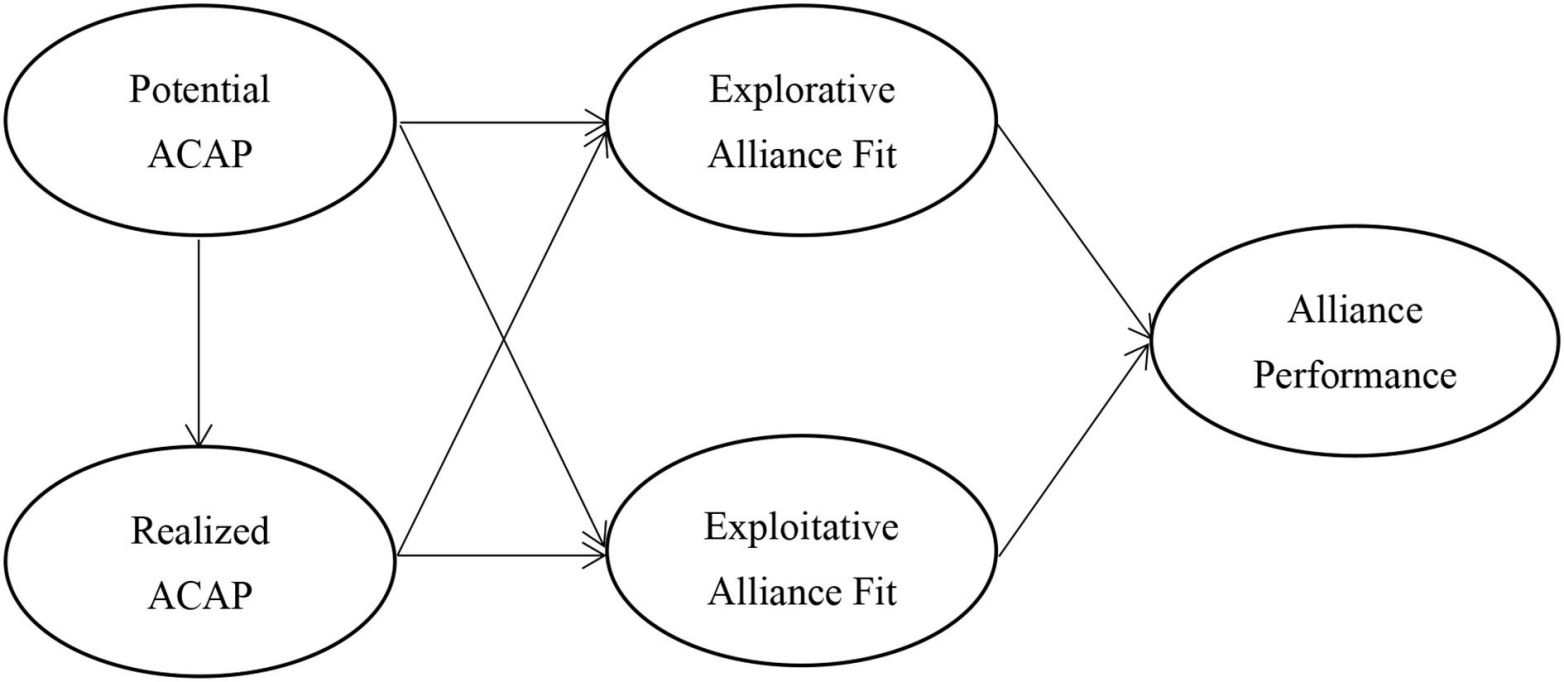
Research framework.

Based on the above hypotheses, this study proposes research framework as Figure 1.

## Methodology

### Sampling

The data were collected through a survey of purposive sampling. The purposive sampling can be implemented based on the individuals' subjective judgment and allows us to select samples that are most suitable for the purpose of the study. The managers of MNEs in Taiwan were taken as the research objects of this study, with an expectation of providing relevant capabilities, experience, and internal scale of production activities through information obtained from such managers. This study takes the 1,000 Taiwanese MNEs as the targets of questionnaire. The questionnaire was mailed to 1,000 MNEs and 219 copies were recalled in actual. Excluding 2 invalid copies, 217 copies were effectively recalled, leading to an effective response rate of 21.7%. Table 1 summaries the respondents' demographic characteristics.

**Table 1:** Demographic characteristics of respondents.

**Characteristics**		**Frequency**	**Ratio**
Industrial sector	Motor manufacturing	83	39.1
	Electronic parts	38	15.2
	Chemicals	23	11.1
	Semiconductors	17	8.0
	Precision machinery	17	8.0
	Information technology	29	13.5
	Other	10	5.1
Profitability	Low profit	105	50.7
	Medium profit	48	19.3
	High profit	63	30.0
Marketing proportion to total costs	Lower than 1%	20	9.4
	1–3%	67	29.1
	3–5%	49	23.2
	5–7%	46	22.0
	More than 10%	35	16.3
R&D proportion to total costs	Lower than 1%	30	11.3
	1–3%	55	26.1
	3–5%	49	23.2
	5–7%	37	17.7
	More than 10%	46	21.7

This study hid the names of constructs and assigned the question items randomly to prevent common method variance (CMV). The Harman one-factor analysis method is used to test CMV. The explained variance in one factor was 39.53%, which is smaller than the recommended threshold of 50%. Therefore, CMV was not problematic in this study (Podsakoff and Organ, 1986). Before conducting hypotheses testing, this study must ensure that the values of the variance inflation factor (VIF) are <5, but the research results showed that the VIF values were between 1.332 and 1.798. Thus, there were no multicollinearity problems among the latent variables (Hair et al., 2017).

### Measures

AC was operationalized as a higher order construct of RAC (transformation and application capacity) and PAC (acquisition and assimilation capacity). The scales of PAC and PAC were measured by Camisón and Forés (2010). These scales consist of nineteen items: acquisition (4 items), assimilation (5 items), transformation (5 items), and application capacity (4 items).

International strategic alliance was defined as enterprises to continuously build and maintain cooperation with the international partners, enabling enterprises to assess foreign markets. Following Rothaermel (2001), Lavie and Rosenkopf (2006), and Nielsen and Gudergan (2012), we employed their measures of ISA: explorative alliance fit (6 items, such as “When selecting your partner for the alliance, how much importance did your company place on access to technology/ knowledge”) and exploitative alliance fit (4 items, such as “How important was the formation of the alliance in allowing access to distribution channels”).

The items related to alliance performance were developed on the basis of the theoretical discussion given in Khalid and Larimo (2012). The final list of five items concerned staying ahead of the partnership, such as “in producing the product for the market when customers needed it through closed partnership”.

### Analysis strategy

This study adopted structural equation modeling to test research hypotheses. For higher order constructs (AC and international strategic alliance), we reduced the parameters to estimate with partial aggregation method (Vinzi et al., 2010). This procedure involves averaging the responses of subsets of items measuring a construct. Because PAC, RAC, and international strategic alliance were multi-dimensional constructs, we average responses of each dimension to serve as indicators for these constructs. Unlike CB-SEM, PLS-SEM is more suitable for this study in the following situations: when the research is conducted for theory development; when the analysis is carried from a prediction perspective; when the structural model is complicated; when one or more formative constructs are included in the structural model; when the sample size is smaller due to a small population; when distribution is lack of normality; and when research requires the latent variable scores for consequent analyses. The above reasons provide supports to consider that PLS is an appropriate SEM method for a study (Chen et al., 2022). Structural validity analysis was performed through SmartPLS v.3.0. Partial least squares structural equation modeling (PLS-SEM) was conducted to verify the structural model.

## Results

### Assessing the measurement model

All scales used in this study were reliable, with Cronbach's α ranging from 0.85 to 0.91 (Table 2). To gauge the reliability and scale validity, we adopted confirmatory factor analysis to verify both the convergent and discriminant validity. In terms of convergent validity, Table 2 shows that the AVE for all dimensions is above the threshold of 0.5 by reference to the criteria proposed by Hair et al. (2010). The CR values of all dimensions are higher than 0.7. Thus, the scale showed a certain convergent validity. The discriminant validity depends on whether the square root of AVE for one dimension is greater than its correlation coefficient with any other dimension(s). As Table 2 shows, this study has discriminant validity.

**Table 2:** Measurement of scales.

	**1**	**2**	**3**	**4**	**5**	**6**	**7**
1. Acquisition	***0.727***						
2. Assimilation	0.540	***0.719***					
3. Transformation	0.472	0.710	***0.754***				
4. Application	0.482	0.711	0.719	***0.716***			
5. Explorative alliance fit	0.307	0.528	0.537	0.527	***0.707***		
6. Exploitative alliance fit	0.342	0.577	0.545	0.523	0.542	***0.758***	
**7. Alliance performance**	0.408	0.513	0.498	0.407	0.453	0.436	***0.758***
Mean	3.525	3.605	3.673	3.622	3.887	3.877	3.808
SD	0.605	0.673	0.617	0.712	0.641	0.713	0.618
α	0.832	0.843	0.814	0.807	0.864	0.843	0.864
AVE	0.529	0.517	0.569	0.513	0.500	0.574	0.574
CR	0.832	0.842	0.814	0.807	0.850	0.843	0.870

The bold values mean squared root of AVEs.

### Test of robustness

To verify the proposed model from being influenced by problems of endogeneity, PLS-SEM was adopted in this study to perform a test of robustness. This study estimated two alternative models (proposed model and competitive model) and compared their model fits. In proposed model, while keeping the other relationships constant, we constructed the first model assuming that PAC and RAC influence explorative alliance fit and exploitative alliance fit, which in turn influences alliance performance. The second model, while also keeping the other relationships constant, assumed that alliance performance influences PAC and RAC, which in turn influences explorative alliance fit and exploitative alliance fit. Most researchers interpret the coefficient of determination (*R*^2^), blindfolding-based cross-validated redundancy measure (*Q*^2^), and standardized root-mean-square residuals (SRMR). As results show, comparison between proposed and competitive models was given that proposed model (*R*^2^ = 0.496; *Q*^2^ = 0.453; SRMR = 0.037) presents better fits than the competitive model (*R*^2^ = 0.328; *Q*^2^ = 0.352; SRMR = 0.082), thus affirming that the proposed model gives a better explanation of the data.

### Test of the structural model

Before the analysis, this study first analyzed the fit of the structural model (Geisser, 1974). Then, Stone-Geisser-Criterion (*Q*^2^), coefficient of determination (*R*^2^), and standardized root-mean-square residuals (SRMR) are used to assess the overall model fit. In our results, *Q*^2^ values were above 0, all *R*^2^ values were more significant than 0.10, and SRMR was < 0.08, meeting the expected criteria (Götz et al., 2010). The VIF values were < 5, ranging from 1.332 to 1.798.

**Figure 2:**
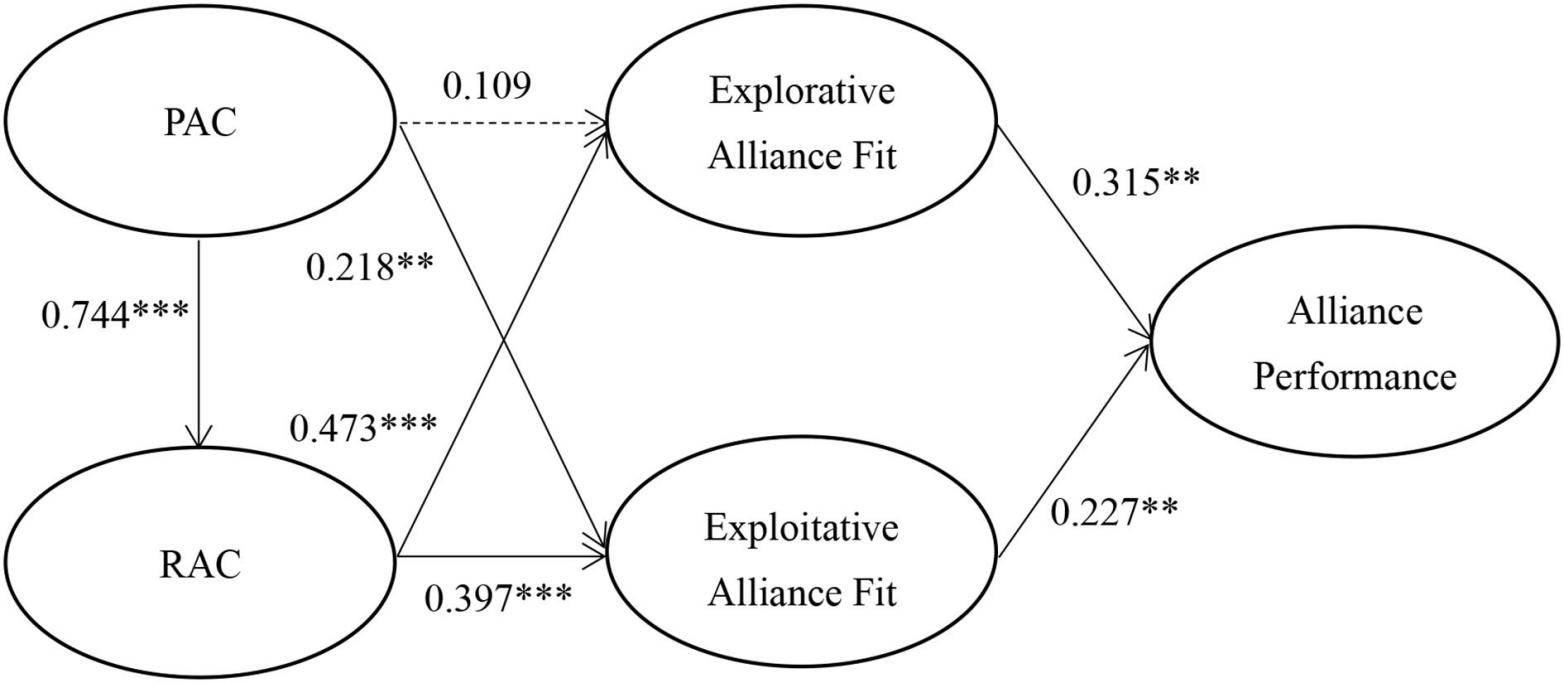
PLSSEM results.

Figure 2 shows the results of the hypothesized relationships and standardized coefficients, including their respective standard errors and *t*-values. This study finds that PAC has a positive effect on exploitative alliance fit (β = 0.218, *p* < 0.01) and RAC (β = 0.744, *p* < 0.001). The results indicate that RAC would be positively associated with explorative alliance fit (β = 0.473, *p* < 0.001) and exploitative alliance fit (β = 0.397, *p* < 0.001); explorative alliance fit would be positively associated with alliance performance (β = 0.315, *p* < 0.01); exploitative alliance fit has a positive effect on alliance performance (β = 0.227, *p* < 0.01); and PAC would not be significantly associated with explorative alliance fit (β = 0.109, *p* > 0.05). Accordingly, H1, H2, H4, H5, H6, and H7 were acceptable and supported except H3. The Stone-Geisser *Q*^2^ values obtained through the blindfolding procedures for RAC (*Q*^2^ = 0.424), explorative alliance fit (*Q*^2^ = 0.387), exploitative alliance fit (*Q*^2^ = 0.377), and alliance performance (*Q*^2^ = 0.453) were larger than zero, supporting the model has predictive relevance (Hair et al., 2017).

### Mediation analysis

The model proposed in this study assumed that explorative and exploitative alliance fit would mediate the relationship among AC (PAC and RAC) and alliance performance. This study tests further for mediation following the approach proposed by Baron and Kenny (1986). PAC (β = 0.263, *p* < 0.01) and RAC (β = 0.212, *p* < 0.05) all demonstrate significant and positive total effects on alliance performance. To further verify meditation mechanisms, as Table 3 shows, this study finds that both PAC and RAC have significant indirect effects on alliance performance and such indirect effects are higher than their direct effects on alliance performance. Moreover, this study additionally adopts several Sobel tests to check whether explorative alliance fit and exploitative alliance fit in PAC and RAC have an influence on alliance performance. The results show that of explorative alliance fit in RAC (*Z* = 2.878, *p* < 0.01) has a significant influence on Sobel t of alliance performance, but not PAC (*Z* = 1.068, *p* > 0.1). Thus, this study concludes that explorative alliance fit partially mediates the effect of AC on alliance performance. Likewise, explorative alliance fit in PAC (*Z* = 1.654, *p* < 0.1) and RAC (*Z* = 2.281, *p* < 0.05) has a significant influence on alliance performance. Thus, exploitative alliance fit fully mediates the effects of PAC and RAC on alliance performance.

**Table 3:** Path coefficient of direct, indirect, and total effects.

**Construct**	**Effects**	**Explorative alliance fit**	**Exploitative alliance fit**	**Alliance performance**
PAC	Direct effect	0.109	0.218	–
	Indirect effect	0.352	0.295	0.279
	Total effect	0.461	0.513	0.279
RAC	Direct effect	0.473	0.397	–
	Indirect effect	–	–	0.239
	Total effect	0.473	0.397	0.239

## Discussion

Our results provide some important insights into the role of international alliance fit and various underlying outcomes of ISAs. Although knowledge transfer and learning through PAC and RAC have become a shot-gun approach for an MNE to access suitable ISAs while improving its own alliance performance that are not easily developed within its confines, the internal competition among alliance partners throws them into dilemmas during knowledge transfer and learning (Rothaermel, 2001; Lavie and Rosenkopf, 2006; Nielsen and Gudergan, 2012). Even if ISAs are highly transparent and open, no one can guarantee that knowledge acquisition is certain to promote the growth of alliance performance. The reason is that MNEs may not have enough knowledge base to acquire and apply the acquired learned knowledge for cooperation in the ISAs context. In this case, as discussed in the embeddedness theory, MNEs need to input resources and cooperate with other members when they join strategic alliances, and as the cooperation deepens, the lock-in effect may be generated, which may cause the loss of investment in exclusive resources (Lin et al., 2018). In this study, based on pervious literatures, several hypotheses were made to explore the relationship among PAC, RAC, ISAs, and alliance performance. Our results provide strong support for research hypotheses with statistical significance. Indeed, almost all the effects of ISAs on the growth of alliance performance exactly direct toward similar features. Other results from the structural model suggest that AC plays an important role in consolidating own knowledge base to strengthen the fit with ISAs, especially through RAC (Peng, 2022; Zhou et al., 2022). This pattern of findings supports organizational learning theorists' view that the fit of international alliance modes concerns the acquisition of different information and knowledge, and has significant impact on the growth of alliance performance (Desai, 2020; Peng, 2022).

### Theoretical implications

This study discusses the relationships between AC, ISAs, and international performance, which have extended recent studies (i.e., Camisón and Forés, 2010; Nielsen and Gudergan, 2012). According to organizational learning and social capital views, this study proposes a complete framework to verify how MNEs leverages external knowledge and existed capability to improve own knowledge base and facilitate it to have more international network-specific knowledge to assess specific ISAs. Specifically, this study provides the following contributions. First, the positive impacts of PAC and RAC on the international explorative and exploitative alliance fit, as stated in previous studies (e.g., Molina-Castillo et al., 2011; Prange and Verdier, 2011; Pinho and Prange, 2016), are verified in an Asian context. This also provides insights into the previous opinions and consolidates the support for the AC and international social network perspectives. However, research finding show that PAC has no statistically significant effect on international explorative alliance fit, that is inconsistent with, and even opposite to research results of Limaj and Bernroider (2019). For closely connected international explorative alliance fit, MNEs should experience a learning process to integrate external knowledge with their knowledge stock, and reinforce positive link between PAC and RAC. In view of the nature of AC and the way that AC develops based on a co-evolutionary approach (Zahra et al., 2006; Limaj and Bernroider, 2019), the findings imply that a well-established PAC facilitates a well-established RAC to strengthen connections with international explorative and exploitative alliance fits. This agrees with the claims of Wang et al. (2015), who particularly pointed out that dynamic capability related to knowledge process (e.g., PAC and RAC) can support each other.

This study takes ISAs as important antecedents to enhance MNEs' international performance, which is different from the research of Liao et al. (2016), Rakthin et al. (2016), Kotabe et al. (2017), and Limaj and Bernroider (2019) that considers AC as antecedent, and contrary to the research findings of Scuotto et al. (2017). Our findings show that explorative and exploitative alliance fits mainly and positively affect international performance, which is related to paradigm of external knowledge acquisition process (Palacios-Marqués et al., 2015a,b). Cohen and Levinthal (1990) argued that firms with AC are able to acquire, assimilate, and transfer external knowledge, and then create novel ideas (Zahra and George, 2002; Flatten et al., 2011; Kang and Lee, 2017; Limaj and Bernroider, 2019). MNEs with stronger PAC and RAC will be easier to access the fit of strategic relationship through more closed connections (Palacios-Marqués et al., 2015a,b), thereby solidifying the foundation of internal knowledge absorption and offering favorable conditions for adaptability in competition.

Further, our results show that explorative and exploitative alliance fits mediate the positive effects of PAC and RAC on international performance, especially through exploitative alliance fit, whilst there are also direct significant positive effects of PAC and RAC on international performance. The findings support the argument that MNEs should build dynamic and closed connections with foreign partners and social network to obtain new knowledge and information about foreign markets and customers to provide certain product and service, as a critically important source of international performance (Harris et al., 2013). Most of previous literatures, as noted earlier, indicated that there are dividing ISAs into different forms (e.g., Lavie and Rosenkopf, 2006; Nielsen and Gudergan, 2012), such as explorative and exploitative alliance fit (Nielsen and Nielsen, 2009; Nielsen and Gudergan, 2012; Li et al., 2013; Ho and Wang, 2015; Pinho and Prange, 2016), strong ties, and weak ties. Our study extends these considerations by specifically confirming the classification of explorative and exploitative alliance fits in the context of MNEs. Moreover, there is an ordinal relation and a reinforcing link between PAC and RAC as mentioned by scholars (Roper et al., 2008; Limaj and Bernroider, 2019). Furthermore, it can be found from structural model that RAC has a higher influence on ISAs than PAC. Not holding the same view with Fosfuri and Tribó's (2008), this study contends that MNEs at a higher level of RAC can gain more revenues from the sales of novel or greatly improved products. This is in line with Zahra and George's (2002) view that RAC is a requisite factor for achieving more excellent international performance.

### Managerial implications

This study has a contribution to the managerial implications concerning how MNEs' AC is correlated with the accessibility to ISAs. When MNEs are able to propose new ideas, improve product features, or develop products preferred by customers, they can build a good relationship with international partners through international strategic alliance and other channels. As a result, managers should involve themselves in maintaining and strengthening ISAs to leverage multiple overseas networks of knowledge for improved international performance.

ISAs have somewhat contributed to the improvement of international performance, but constraints gradually show up as international markets expand. A sufficient number of knowledge bases are perquisite for MNEs to collect intelligence from ISAs and clearly understand them.

Cadogan et al. (2003, 2009) proposed the concept of international market orientation. In this context, PAC and RAC are still critical factors to have a better understanding of the international market by means of collection and application of international information. Firms need to interpret international information using their existing PACs and RACs. Furthermore, managers are recommended in this study to develop PAC and RAC through formal and informal knowledge-processing mechanisms. Examples of formal knowledge-processing mechanisms are integrated information system, electronic communication system, Internet and business intelligence, etc., while informal knowledge-processing mechanisms include inter-departmental personal relationships, banquets, information-sharing social network, and among others.

### Research limitations and suggestions for future studies

Despite the existence of previous studies which have discussed much about antecedents of dynamic capabilities, the driving factor for discussing MNEs in the international context is under-researched. In this study, the formulation of AC is explored and significant insights are proposed through ISAs. Nevertheless, there are still some limitations in the study. First, from the perspective of international relationships, this study proposes two different categories of ISAs. Although both kinds of ISAs are beneficial to the cultivation of international exploration and exploitation, it fails to verify which kind(s) of ISAs are more beneficial to the development of AC. However, the participation of various ISAs requires corresponding costs or specific investment, which can be a heavy burden for MNEs. Thus, it is suggested that the influence of different ISAs on AC can be further compared in subsequent studies, and from among which, the best input and participation can be selected.

Furthermore, in addition to ISAs, there are still many variables influencing the formulation of AC. Different theories added into the research framework may result in different outcomes, such as knowledge transfer perspective, organizational learning theory, innovation theory, etc. This study suggests that researchers can add more valuable variables through the dialogue between different theories to enrich the internationalization theory. Thirdly, the research sample in this study focuses on MNEs. However, in the process of internationalization, it is possible that many MNEs may improve the scale due to the expansion of market or the growth of operation, and many studies have indicated that enterprises of different scales may differ in strategic behaviors. Thereby, this study suggests that in addition to taking large enterprises as research samples, future researchers can also compare the differences between enterprises in different countries and add cross-cultural differences to make the study more valuable for reference.

## Conclusion

This study aims to discuss the effect of MNEs' PAC and RAC on international strategic alliance from the angle of organizational learning and embeddedness theories, and further examines the development of alliance performance. The results show that RAC plays a key role in the research framework and has a positive effect on international explorative and exploitative alliance fits, which is reflected in subsequent alliance performance. The research findings offer more implications in the organizational learning theory and enrich the embeddedness theory. However, there are no more longitudinal profile data to support the further study on the dynamic development of AC and ISAs from the view of dynamic capability. Thus, it is hoped that researchers can discuss this subject in their future studies.

## Data availability statement

The raw data supporting the conclusions of this article will be made available by the authors, without undue reservation.

## Ethics statement

The studies involving human participants were reviewed and approved by Academic Committee of Foshan University. The patients/participants provided their written informed consent to participate in this study.

## Author contributions

ZC, LZ, and MP contributed to conception and design of the study and wrote sections of the manuscript. LS organized the database. MP performed the statistical analysis and wrote the first draft of the manuscript. All authors contributed to manuscript revision, read, and approved the submitted version.

## Conflict of interest

The authors declare that the research was conducted in the absence of any commercial or financial relationships that could be construed as a potential conflict of interest.

## Publisher's note

All claims expressed in this article are solely those of the authors and do not necessarily represent those of their affiliated organizations, or those of the publisher, the editors and the reviewers. Any product that may be evaluated in this article, or claim that may be made by its manufacturer, is not guaranteed or endorsed by the publisher.
